# Nanomolecularly-induced Effects at Titania/Organo-Diphosphonate Interfaces for Stable Hybrid Multilayers with Emergent Properties

**DOI:** 10.1021/acsanm.4c00743

**Published:** 2024-05-03

**Authors:** Collin Rowe, Ankit Kashyap, Geetu Sharma, Naveen Goyal, Johan G. Alauzun, Seán T. Barry, Narayanan Ravishankar, Ajay Soni, Per Eklund, Henrik Pedersen, Ganpati Ramanath

**Affiliations:** † Materials Science & Engineering Department, Rensselaer Polytechnic Institute, Troy, New York 12180, United States; ‡ School of Physical Sciences, Indian Institute of Technology Mandi, Mandi, Himachal Pradesh 175005, India; § Materials Research Centre, Indian Institute of Science, Bangalore, Karnataka 560012, India; ∥ Institut Charles Gerhardt, University of Montpellier, CNRS, ENSCM, 34293 Montpellier, France; ⊥ Department of Chemistry, Carleton University, Ottawa, Ontario K1S 5B6, Canada; # Department of Physics, Chemistry, and Biology, Linköping University, SE-58183 Linköping, Sweden

**Keywords:** inorganic−organic hybrid materials, thin film growth, multilayers, atomic layer deposition, molecular layer deposition, molecular nanolayer, morphology

## Abstract

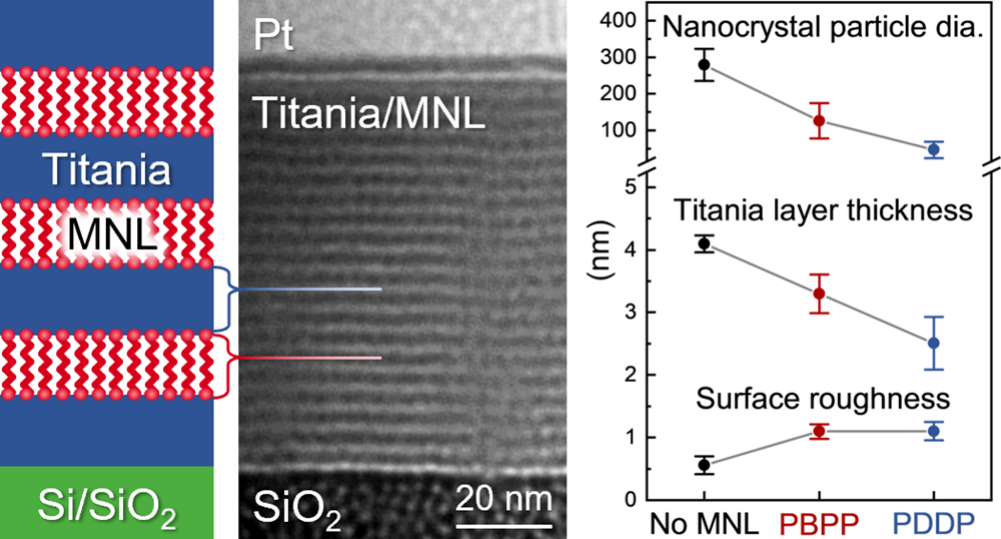

Nanoscale hybrid inorganic–organic multilayers are attractive for accessing emergent phenomena and properties through superposition of nanomolecularly-induced interface effects for diverse applications. Here, we demonstrate the effects of interfacial molecular nanolayers (MNLs) of organo-diphosphonates on the growth and stability of titania nanolayers during the synthesis of titania/MNL multilayers by sequential atomic layer deposition and single-cycle molecular layer deposition. Interfacial organo-diphosphonate MNLs result in ∼20–40% slower growth of amorphous titania nanolayers and inhibit anatase nanocrystal formation from them when compared to amorphous titania grown without MNLs. Both these effects are more pronounced in multilayers with aliphatic backbone-MNLs and likely related to impurity incorporation and incomplete reduction of the titania precursor indicated by our spectroscopic analyses. In contrast, both MNLs result in two-fold higher titania nanolayer roughness, suggesting that roughening is primarily due to MNL bonding chemistry. Such MNL-induced effects on inorganic nanolayer growth rate, roughening, and stability are germane to realizing high-interface-fraction hybrid nanolaminate multilayers.

## 1 Introduction

Molecular nanolayers (MNLs) at inorganic interfaces can enhance a variety of properties^1^ such as interfacial strength,^2^ electrical and thermal transport,^3^ and diffusion barrier performance^4,5^ for electronic and energy applications. Stacking inorganic/MNL interfaces offers possibilities for accessing emergent properties by superposition effects from proximal and distal interfaces.^1^ Our prior works indeed support the emergence of unexpected properties, such as loading-frequency-dependent interfacial toughening to levels beyond the static-load toughness^6^ and viscoelastic band gaps.^6,7^ Nanoscale multilayering of inorganic/MNL interfaces also allows the possibility of inducing properties such as those seen in nacre and bone,^8^ but via smaller length scale phenomena that are not constrained by biological processes. Such properties are attractive for diverse applications including microelectro-mechanical-systems,^9^ self-healing/destructing materials,^10,11^ and transparent conductors.^12^ Syntheses of such high-interface-fraction hybrid nanomaterials also pave the way for a new class of materials whose properties are primarily dictated by the inorganic/MNL structure and chemistry.

Metal-oxide-based hybrid thin film multilayers have been fabricated^13^ by sequential atomic layer deposition (ALD) and molecular layer deposition (MLD). Such nanolaminates exhibit unusual property combinations different from that of the constituent materials, e.g., high electrical^12^ and low thermal conductivities,^14,15^ and mechanical flexibility.^16,17^ However, very little is known about the effects of the MNL structure and chemistry on the nano/micro-scale structure, morphology, and phase stability of inorganic nanolayers, as well as the inorganic/MNL interfaces.^18^

**Figure 1:**
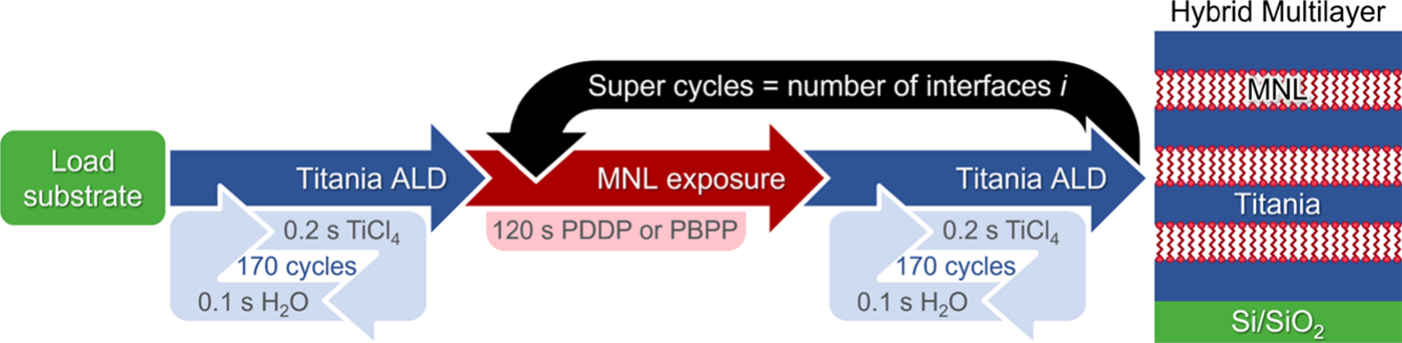
Scheme for synthesizing titania/organo-diphosphonate MNL multilayers by sequential titania ALD and single-cycle MNL single-cycle molecular exposures of diethyl-(12-phosphonododecyl) phosphonate (PDDP)—an aliphatic diphosphonate , or tetraethyl-[1,1′-biphenyl]-4,4′-diylbis-phosphonate (PBPP)—an aromatic diphosphonate.

Here, we report the synthesis of titania/organophosphate-MNL multilayers (see Scheme in Figure 1) and describe MNL-induced decreases in the titania nanolayer growth rate, suppression of anatase nanocrystal formation, and increases in surface roughness. We show that these phenomena are related to the MNL structure, bonding chemistry, and impurity incorporation during titania growth. These findings provide insights into MNL-induced effects on the growth, structure, and stability of the inorganic layers for the design of inorganic–organic hybrid nanolayer nanolaminates.

Our choice of organophosphonate MNLs is motivated by their multifold increase in interfacial adhesion and thermal conductance.^3,19^ Phosphonate groups are typically stable up to around 800 °C,^20,21^ and the organo-diphosphonates used here are insensitive to moisture (e.g., unlike organosilanes^22,23^), which are conducive for easy handling. Organophosphonates form dense monolayers^24^ on a variety of oxide surfaces (e.g., TiO_2_, Al_2_O_3_) via primarily bi- and tri-dentate P–O-M bridges^22,25^ to impact diverse properties.^26−30^ While organophosphonate formation on inorganic materials has been studied extensively, the reverse, i.e., formation of inorganic thin films or nanoparticles on organophosphonates, is yet to be explored. Prior works have created surface MNLs and *single* inorganic/MNL/inorganic interfaces through wet-chemical self-assembly^26,31−33^ of organophosphonates. Here, we combine ALD of titania and single-cycle MLD of organophosphonate MNLs to synthesize *multilayered* stacks of titania/MNL/titania interfaces and study the effects of MNLs on the inorganic nanolayers. Single-cycle MLD with one precursor favors the assembly of an organic MNL via phosphonate-oxide bonding instead of polymeric layers obtained by successive cyclic pulses of more than one precursor through surface-limited reactions (e.g., click chemistry).^34,35^

## 2 Results and Discussion

### 2.1 Organophosphonate and Titania Nanolayer Deposition

**Figure 2:**
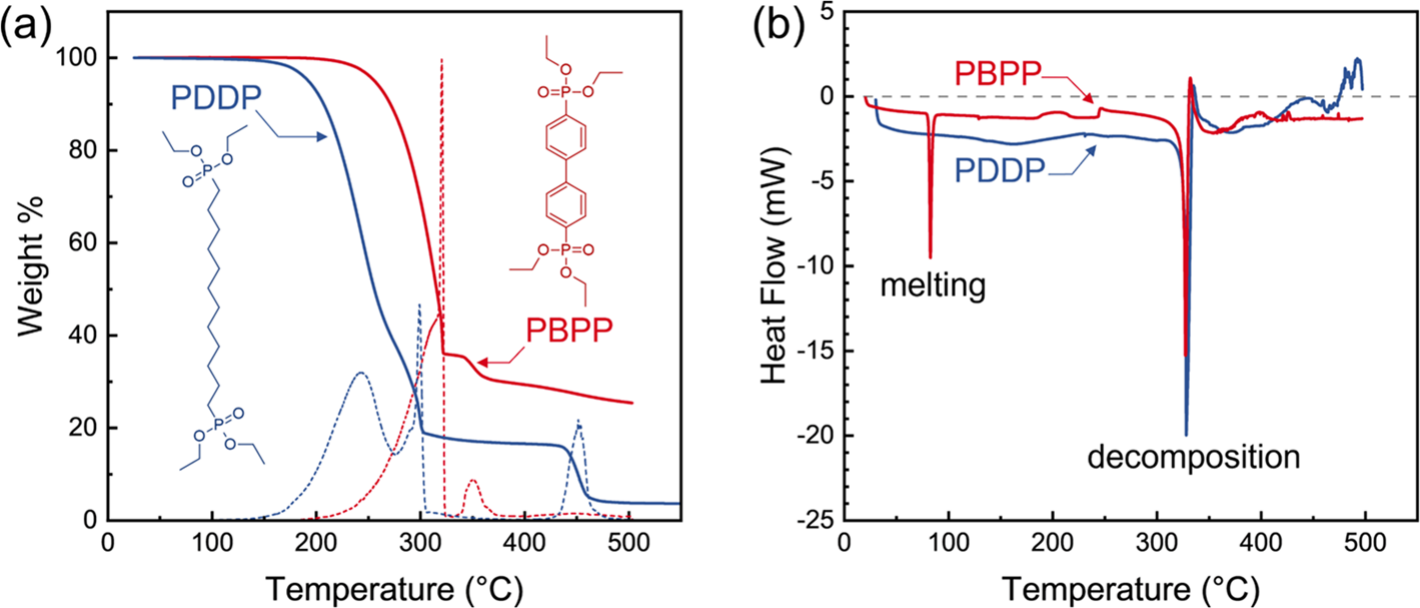
(a) TGA characteristics from PDDP (blue, bold) and PBPP (red, bold) shown along with their first derivatives (dotted lines). (b) DSC curves from PDDP (blue) and PBPP (red), with the dashed horizontal line denoting zero heat flow change.

We studied two diphosphonate MNLs with identical terminal moieties and number of carbon atoms but with different backbone structures. The aliphatic backbone diphosphonate is diethyl-(12-phosphonododecyl) phosphonate, henceforth referred to as PDDP. The aromatic backbone diphosphonate is tetraethyl-[1,1′-biphenyl]-4,4’diylbis(phosphonate), henceforth referred to as PBPP. PDDP melts around room temperature and PBBP melts at ∼75 °C. Thermogravimetric analysis (TGA) shows that PDDP starts to evaporate at ∼150 °C followed by a complex thermolysis with overlapping evaporation and decomposition (Figure 2a). Similarly, PBPP starts to evaporate at ∼200 °C, followed by a complex thermolysis. In both cases, the decomposed molecules leave behind residual masses. Differential scanning calorimetry (DSC) scans from both molecules exhibit endotherms leading into exotherms around ∼300–325 °C (Figure 2b), indicative of complex thermolysis and decomposition.^36^ Accordingly, we set both the MNL precursor ampule temperature and the ALD/MLD reaction chamber temperature to 180 °C, which allows a small vapor pressure conducive to MNL formation and precludes MNL decomposition during subsequent titania overlayer depositions.

**Figure 3:**
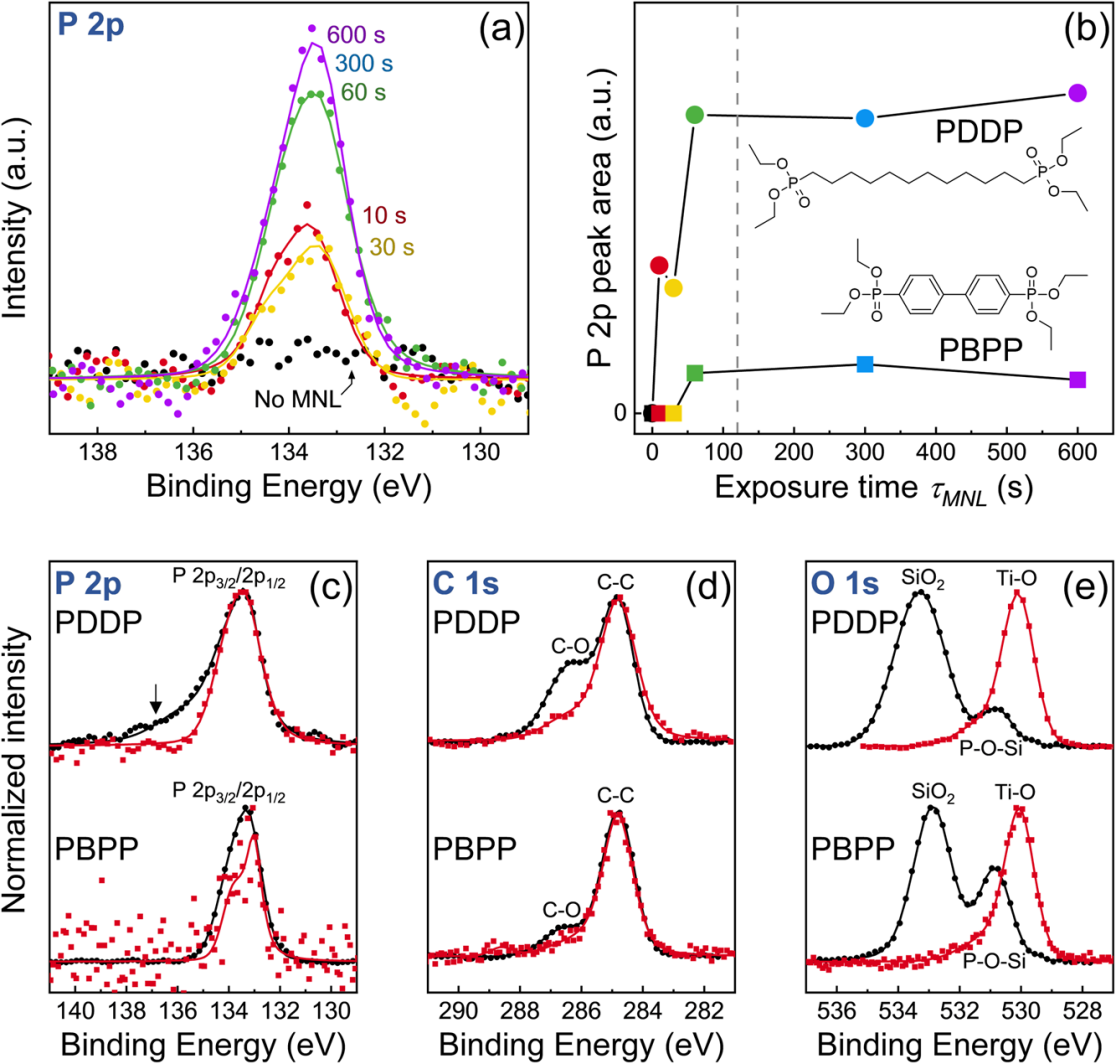
(a) XPS spectra around the P 2p peak from titania surfaces exposed to PDDP for different times τ_MNL_. (b) P 2p peak areas plotted vs τ_MNL_ capturing PDDP and PBPP MNL formation. The vertical dashed line denotes the τ_MNL_ chosen for all our multilayer depositions. XPS spectra around (c) P 2p, (d) C 1s, and (e) O 1s peaks from titania surfaces exposed to PDDP (top) or PBPP (bottom) for τ_MNL_ = 60 s (red squares). Spectra from as-received PDDP and PBPP on Si are shown for comparison (black circles). The arrow in (c) points to the overlap between Si 2p satellite peak and the P 2p region.

X-ray photoelectron spectroscopy (XPS) showing P 2p signatures on titania surfaces confirms that 180 °C is sufficient to form PDDP and PBPP MNLs on titania (Figure 3a). The optimal organophosphonate pulse time τ_MNL_ was determined by exposing ∼100-nm-thick titania films to PDDP or PBPP for 10 s ≤ τ_MNL_ ≤ 600 s (Figure 3b). For both PDDP and PBPP, the P 2p peak intensities saturate at τ_MNL_ ≥ 60 s. Inferring this to be indicative of MNL formation, we used τ_MNL_ = 120 s for obtaining interfacial MNLs for both chemistries to ensure sufficient surface coverage.

XPS spectra from neat PDDP and PBPP on silica surfaces as well as their MNLs on titania surfaces exhibit P 2p peaks at 133.5 ± 0.2 eV corresponding to phosphonate moieties^37^ (Figure 3c). The C–O sub-bands around 286.4 eV seen in spectra^38^ from neat PDDP and PBPP are negligible or undetectable in spectra from the corresponding MNLs on titania for τ_MNL_ = 60 s (Figure 3d). This result suggests that MNL formation involves cleavage of ethoxy groups in the organophosphonates and covalent P–O–Ti bridging with the titania surface.^25^ Neat PDDP and PBPP molecules on silica exhibit O 1s sub-bands from phosphonate at 530.8 eV, which are at least partly associated with P–O–Si anchoring bonds,^39^ as well as SiO_2_ at 533.0 eV (Figure 3e), with a smaller phosphonate/SiO_2_ ratio for neat PDDP than for neat PBPP. Titania/MNL multilayers, with either PDDP or PBPP, exhibit a shoulder at 531.3 eV, likely arising from phosphonates including P–O–Ti anchoring bonds,^37,40^ and/or surface moisture.

We carried out titania ALD^41,42^ also at 180 °C with 0.2 and 0.1 s pulses of TiCl_4_ and H_2_O, respectively. XPS analyses reveal an average Ti/O ratio of 0.42 ± 0.02, indicating O-rich film surfaces compared to stoichiometric TiO_2_. There are no discernible trends of Ti, O, and C content with precursor exposure τ_MNL_ (see Figure S1).

### 2.2 Titania/Organophosphonate Multilayers

Titania/organophosphonate MNL multilayers were deposited on Si(100) substrates with ∼300 nm of thermally grown wet SiO_2_ by sequential ALD of titania and single-cycle MLD of PDDP or PBPP MNLs at a 7 mbar pressure using the scheme in Figure 1. Each titania layer was obtained from 170 ALD cycles. In all the multilayers, the first and the last layers are titania. Thus, if *i* denotes the number of interfaces, *i* + 1 is the number of titania layers. Titania films were deposited with identical ALD cycles but *without* MNLs to serve as a baseline for assessing MNL-induced effects.

**Figure 4:**
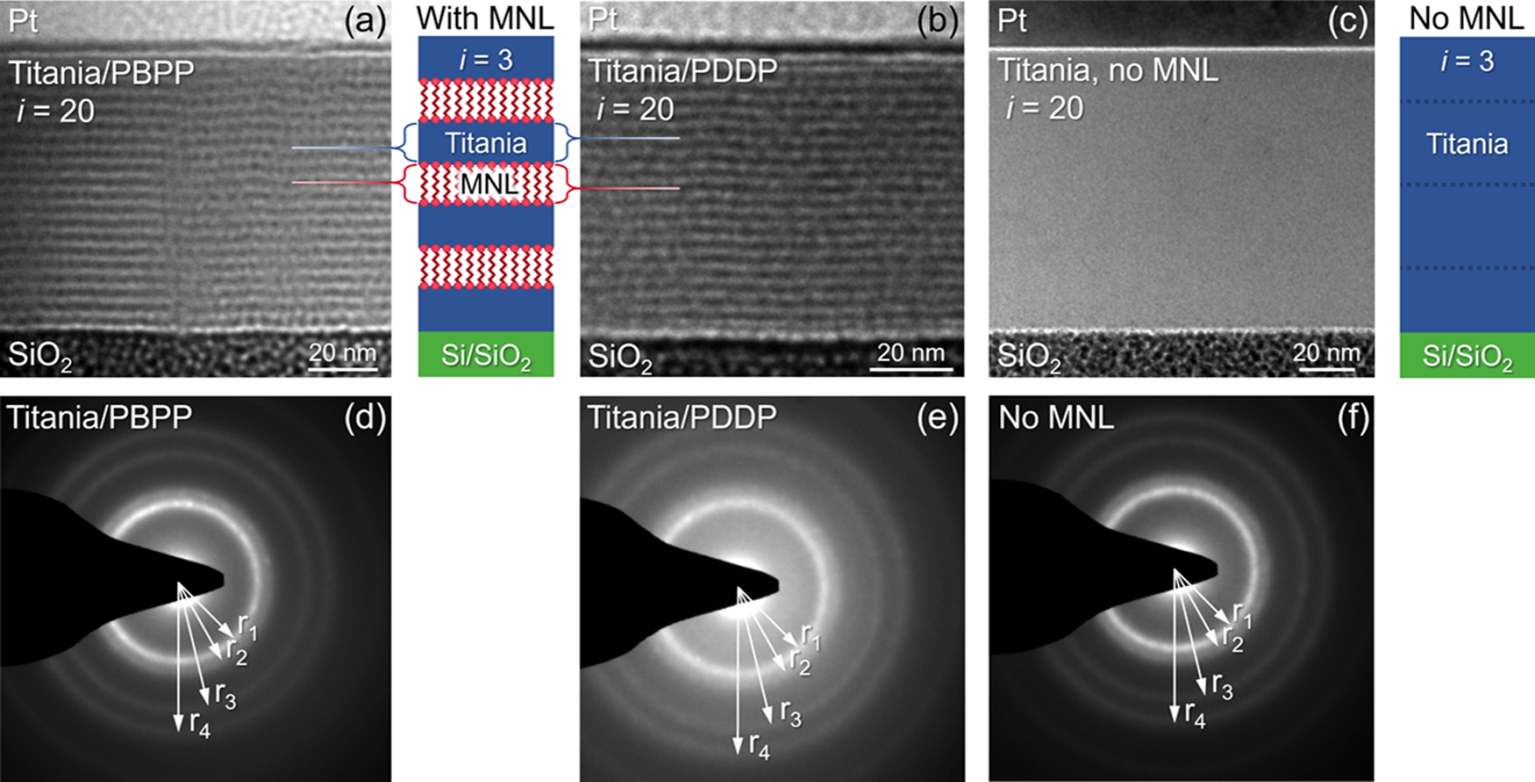
Cross-section TEM images from titania/MNL multilayers with *i* = 20 for (a) PBPP and (b) PDDP MNLs, and (c) titania without MNLs. Schematics show structures with *i* = 3; dashed lines in structures without MNLs denote 170 cycle ALD intervals. Electron diffractograms from (d) titania/PBPP and (e) titania/PDDP multilayers and (f) titania film without MNL.

Cross-sectional bright-field transmission electron microscopy (TEM) images from titania/MNL nanolaminates (Figure 4a,b) with *i* = 20 show multilayering with discrete titania layers (light) separated by organophosphonate MNL (dark). Grainy contrast in films with MNLs is due to electron beam damage, as expected. The contrast deteriorates with extended electron exposure. This effect is not seen in titania films without MNLs (Figure 4c). Broad diffuse rings seen in diffractograms from titania films with and without MNLs are indicative of amorphous titania (Figure 4d–f). The rings encompass Bragg peaks associated with anatase titania, suggesting the presence of anatase-like short-range order in the amorphous titania nanolayers. For instance, the first ring r_1_ corresponds to (103), (004) and (112); the second ring r_2_ to (200); the third r_3_ to (204), (116) and (220); and the fourth r_4_ to (215) and (303) (see Figure S2).

**Figure 5:**
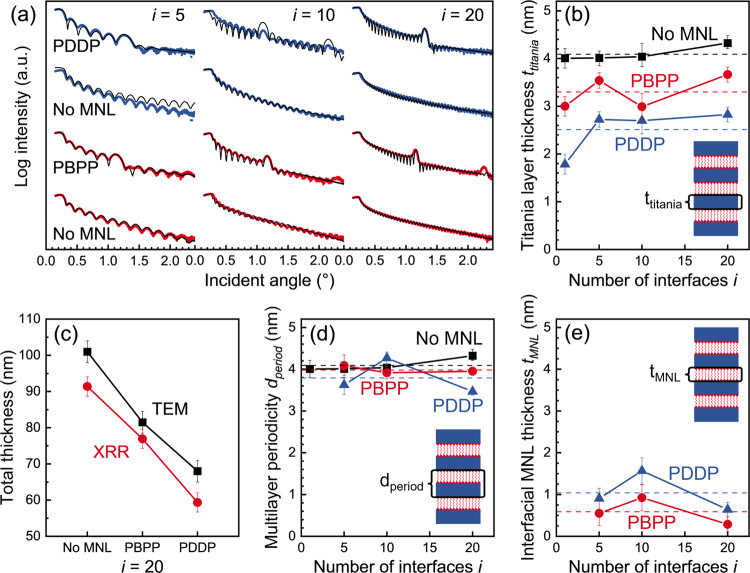
(a) XRR plots from titania/PBPP (blue) and titania/PDDP (red) nanolaminates, with *i* = 5, 10, and 20, and model fits (black), shown along with plots from titania films without interfacial MNLs. (b) Individual titania layer thickness *t*_titania_ determined from the Kiessig fringes. (c) MNL-induced decrease in titania nanolayer thickness for *i* = 20 from XRR and TEM. (d) Multilayer periodicity *d*_period_ from XRR Bragg peaks. (e) MNL thickness *t*_MNL_. Dashed lines in (b), (d), and (e) denote averages for each data set.

X-ray reflectivity (XRR) measurements from multilayered titania/organophosphonate interfaces exhibit Kiessig fringes as well as Bragg peaks indicating one-dimensional periodicity parallel to the substrate normal (Figure 5a). Fitting the Kiessig fringes with simulations^43^ for model titania/MNL multilayers and titania films without MNLs yields the total film thickness *t*_total_ from which we determined the average titania nanolayer thickness *t*_titania_ = *t*_total_/(*i* + 1). For the same *i*, MNL-interfaced multilayers exhibit a lower *t*_titania_ than titania films without MNLs (Figure 5b), indicating that the titania growth per cycle rate is lower on organophosphonate MNLs than on titania. Film thicknesses measured by cross-sectional TEM after aligning the Si(001) substrate along the [110] zone axis confirm this trend, with a systematic offset of about 0.6 nm (Figure 5c). The MNL-induced decrease in titania nanolayer growth per cycle is more pronounced for PDDP at ∼39% than for PBPP at ∼19% when compared to that of titania films with no MNLs.

In contrast to the above, the Bragg peaks seen at θ = 1.2° ± 0.1° are identical for multilayers with PDDP and PBPP MNLs, indicating a multilayer periodicity *d*_period_ = 3.9 ± 0.3 nm (Figure 5d) as determined from a modified Bragg’s law (see Supporting Information). Regarding *d*_period_ = *t*_titania_ + *t*_MNL_ for titania/MNL multilayers, we estimate *t*_MNL_ = 0.8 ± 0.4 nm (Figure 5e), which is consistent with a single layer of PDDP or PBPP. For titania films without MNLs, we consider *t*_titania_ = *d*_period_.

Since both molecules have the same terminal moieties, the slower growth of titania on the MNLs must be related to MNL morphology and coverage, which are sensitive to the molecular backbone structure. During ALD on pristine titania surfaces, titania nanolayers form by reaction of TiCl_4_ with surface hydroxyl groups.^41^ MNL formation through coupling between phosphonate moieties and hydroxyl groups renders the unanchored ethoxy groups on the other end of the disphosphonate molecules as active sites for the TiCl_4_ reaction.^44^ The slower kinetics^45^ of the TiCl_4_-ethoxy reaction and the inherently lower coverage of unanchored ethoxy groups compared to the hydroxyl density on pristine titania are likely contributors for the MNL-induced decrease in titania nanolayer growth per cycle.

Our XPS results showing a higher P concentration upon saturation of titania surfaces with PDDP (Figure 3b) than PBPP indicate a higher coverage of PDDP on titania. If we assume that one phosphonate group in each molecule of the organophosphonate MNLs is anchored to the substrate and the other is available for TiCl_4_ reaction, the observed result would imply that PDDP provides a higher density of active ethoxy moieties for titania formation than PBPP. This, however, is contrary to the greater effect of PDDP (than PBPP) in slowing down the titania nanolayer growth kinetics. We are thus persuaded to infer that our organophosphonate MNLs are not assemblies of highly oriented molecules. For example, the more flexible aliphatic backbone in PDDP MNLs could result in both phosphonate termini of a given molecule to anchor to the substrate, which would actually decrease the active ethoxy moieties available for TiCl_4_. These findings suggest that while MNL surface coverage and molecular morphology are sensitive to the backbone structure, these factors could have opposing effects on the titania nanolayer growth kinetics. Systematic studies of the effects of molecular morphology, orientation, and surface coverage on the kinetics of inorganic overlayer formation are necessary to uncover and understand these effects.

**Figure 6:**
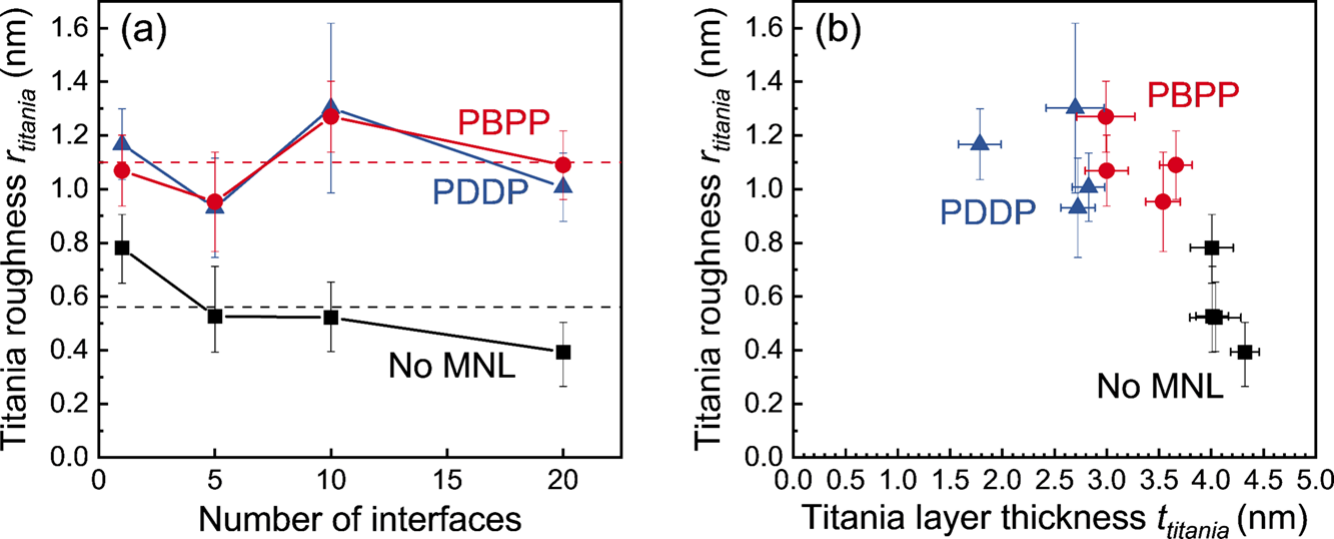
Titania nanolayer roughness *r*_titania_ plotted versus (a) the number of interfaces *i*, and (b) the titania thickness *t*_titania_. The dashed lines in (a) indicate the average *r*_titania_ values for multilayers with (red) and without (black) MNLs.

Model fits of XRR data for a given *i* show that titania/MNL multilayers with either PBPP or PDDP show a two-fold higher root-mean-square roughness *r*_titania_ (Figure 6a) than titania films without MNLs. This result suggests that MNL-induced titania nanolayer roughening is governed by the phosphonate terminal chemistry rather than the backbone structure of the molecules comprising the MNL. This inference is supported by the lack of correlation between MNL-induced roughening and titania nanolayer thickness, which is sensitive to the organophosphonate backbone structure (Figure 6b). These results collectively indicate that MNL-induced roughening is primarily related to the titania overlayer/MNL interface chemistry.

### 2.3 Stoichiometry, Density, and Impurity Incorporation

**Figure 7:**
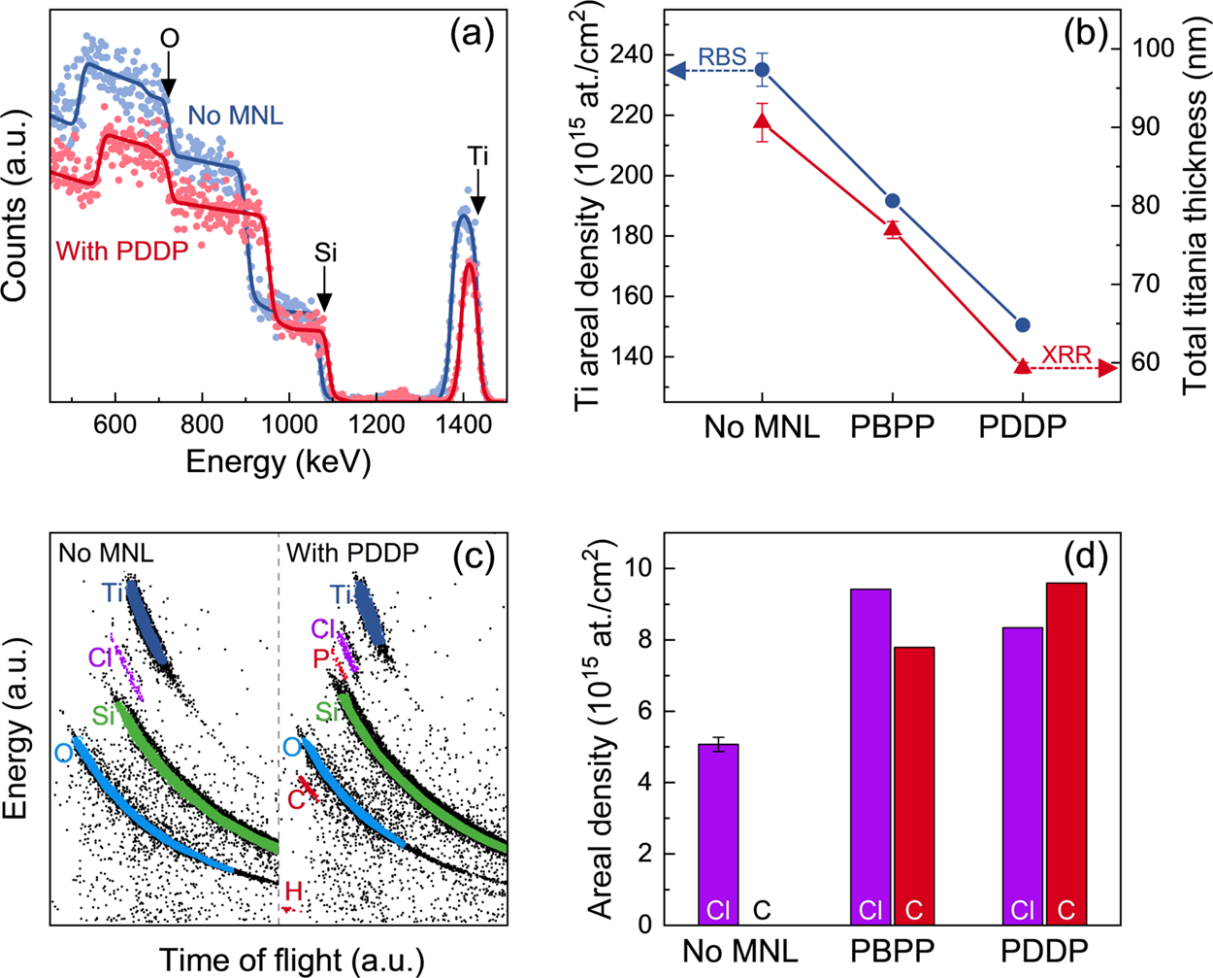
(a) RBS spectra (points) and SIMNRA^32^ simulations (lines) from *i* = 20 titania/PDDP MNL multilayers and monolithic titania films without MNL. (b) Ti areal densities for titania multilayers for *i* = 20 with no MNLs, and with PDDP or PBPP MNLs, determined from RBS (blue). The total titania thickness from XRR (red) is used to determine the bulk density of titania. (c) ERDA data from *i* = 20 titania/PDDP MNL multilayers and titania films without MNLs overlaid with Potku^33^ simulations for different elements. (d) Areal densities of Cl and C determined by ERDA for titania multilayers with no MNLs, and with PDDP or PBPP MNLs.

Rutherford backscattering spectroscopy (RBS) and elastic recoil detection analysis (ERDA) corroborate the MNL-induced decreases in titania growth rates. SIMNRA^46,47^ fits of RBS spectra indicate a 18% lower Ti peak intensity in titania/PBPP multilayers than titania films without MNLs deposited under identical conditions. Titania/PDDP multilayers show a 36% lower intensity than titania films without MNLs (Figure 7a). Potku^48,49^ simulations of ERDA data corroborate these trends, which are consistent with the MNL-induced decreases in titania growth rate indicated by XRR and TEM.

RBS and ERDA show an average bulk Ti:O stoichiometry of 0.50 ± 0.03 for films with and without MNLs. Dividing the Ti areal densities from RBS with the titania thicknesses obtained from XRR (Figure 7b), we obtain a titania bulk density of ∼3.5 g/cm^3^ for multilayers with and without MNLs. Thus, organophosphonate MNLs have a negligible effect on the composition and density of the amorphous titania nanolayers grown on them.

ERDA of films with MNLs also show P, C, and H signals expected from organophosphonates; these are not seen in titania films without MNLs (Figure 7c). Titania/MNL multilayers show 60–90% higher Cl content than films without MNLs (Figure 7d). XPS analyses of the Cl 2p peaks also show higher traces of Cl on surfaces of titania/MNL multilayers than on titania films without MNLs, but the low signal-to-noise ratios preclude quantification. These results suggest that titania ALD on organophosphonate MNLs may be due to increased incomplete half-reactions of TiCl_4_ and/or Cl trapping at the MNL interface (e.g., chlorination of aromatic moieties or formation of P–Cl bonds). These intriguing results suggest that an MNL can unobtrusively but profoundly impact the reaction path and kinetics of inorganic overlayer deposition, which are worthy of further investigation due to their relevance for other materials systems.

### 2.4 Phase and Morphological Stability

**Figure 8:**
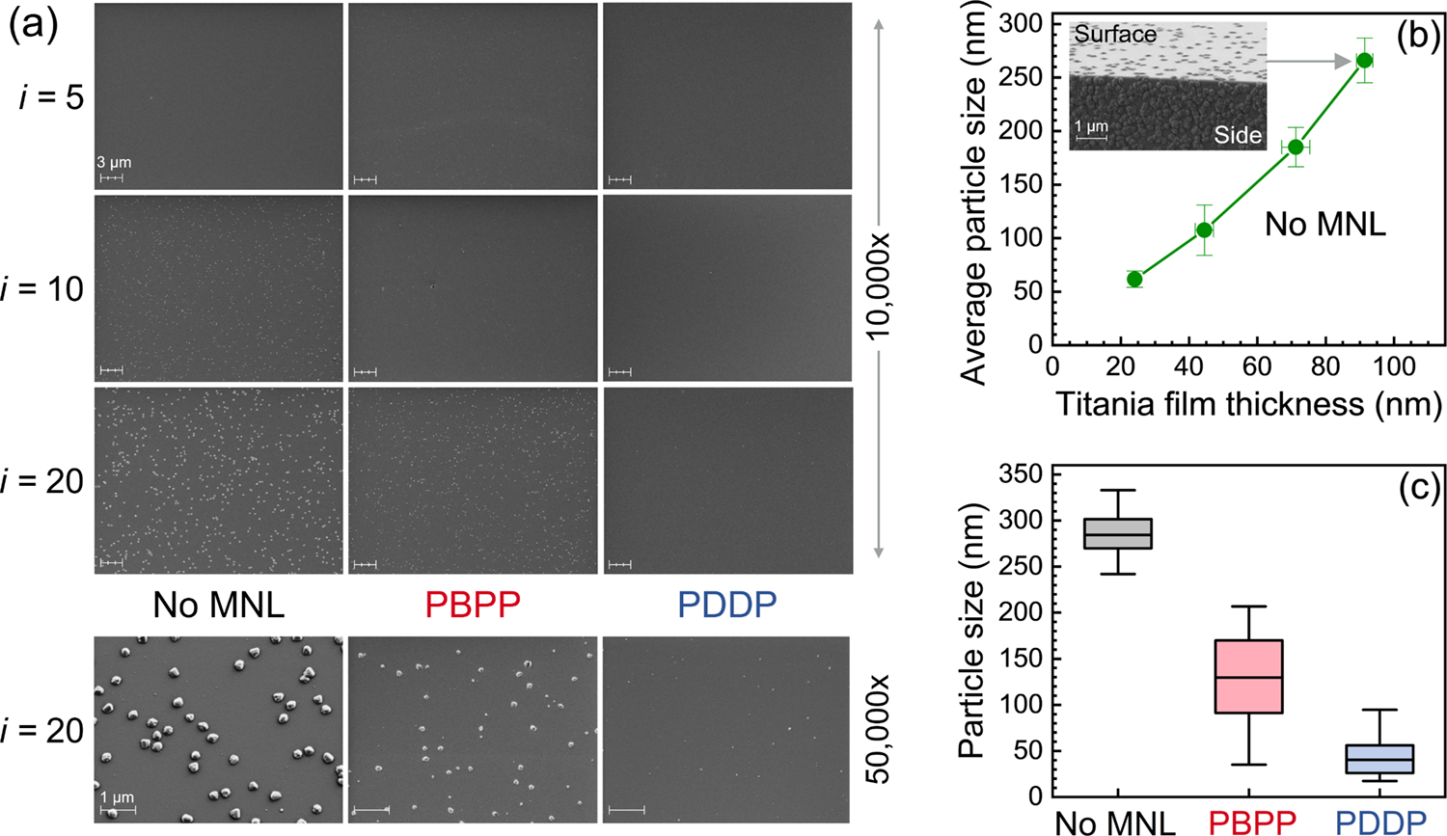
(a) SEM images from surfaces of titania films with and without interfacial MNLs for different numbers of interfaces *i*. (b) Average nanoparticle diameter as a function of the titania film thickness for films without MNLs. Inset SEM image with a stage tilt of 70° showing the particles forming from the deposited titania film on the surface and sides of the substrate. (c) Box and whisker plot of the size distribution of 100 surface particles for titania films with *i* = 20 with interfacial PDDP or PBPP MNLs, and a titania film with no MNLs.

Analyses of SEM micrographs from titania multilayers with and without interfacial MNLs indicate that both PDDP and PBPP MNLs inhibit titania nanoparticle formation (Figure 8a). For *i* ≥ 5, we observe nanoparticles in ALD titania films grown *without* MNLs. SEM images obtained immediately after deposition also show nanoparticles, indicating that nanoparticle formation occurs during ALD. The titania particle size increases with increasing film thickness (Figure 8b), which is significantly greater than the titania nanolayer thickness. For example, the average particle size *p* increases from ≈ 60 to ≈ 270 nm as the number of interfaces (without MNLs) is increased from *i* = 5 to *i* = 20.

Introducing either PDDP or PBPP interfacial MNLs suppresses nanoparticle formation with PDDP showing a greater suppression. In titania/MNL multilayers, the nanoparticles are smaller and have a broader relative size distribution around the average than nanoparticles seen in titania films of similar thicknesses without MNLs (Figure 8c). The sensitivity of the nanoparticle size and distribution to titania film thickness and the presence of MNLs indicate that these nanoparticles nucleate from the amorphous titania nanolayer and are unlikely to be formed in the gas phase.

**Figure 9:**
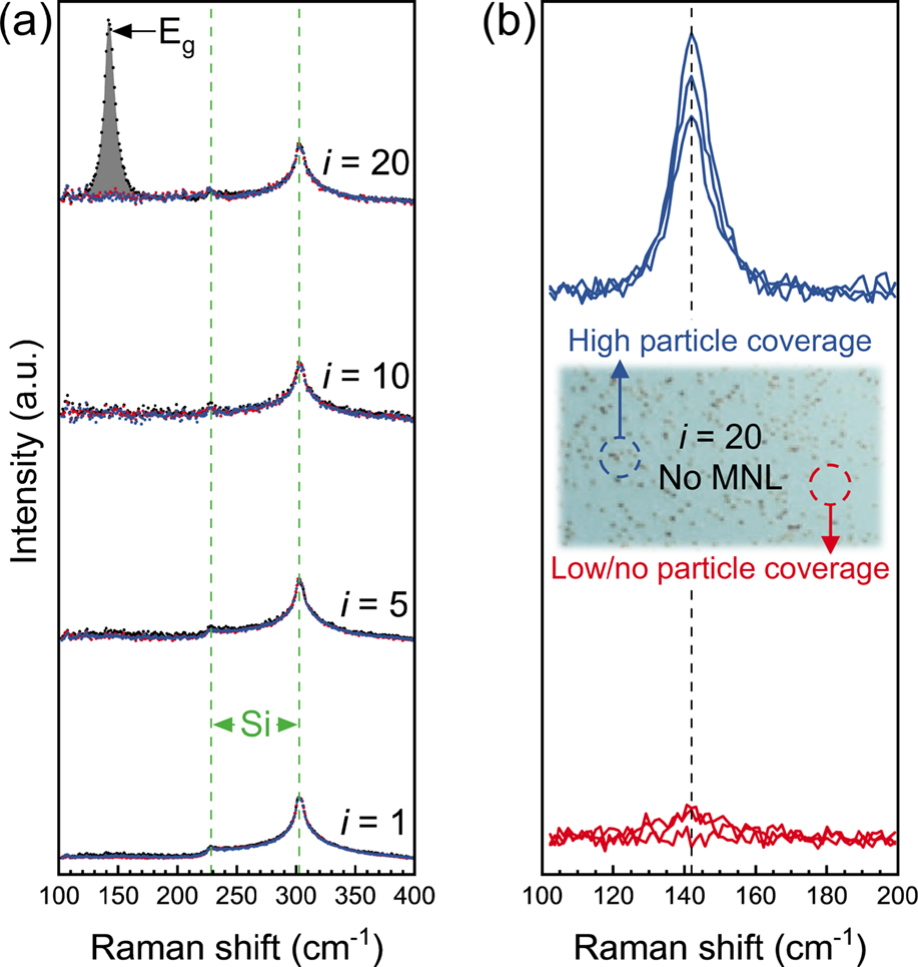
(a) Raman spectra from titania films with interfacial PBPP (red) or PDDP (blue) MNLs, and no MNLs (black), for different number of interfaces *i*. (b) Raman spectra from titania film (*i* = 20) without interfacial MNLs at regions of high and low/no nanoparticle coverage. Inset shows an optical image indicating example regions of high and low particle coverage from where the spectra were collected.

Raman spectra indicate that the titania nanoparticles are comprised of the anatase phase. The silica substrate Raman peak at ∼302 cm^–1^ is seen in all titania/MNL and titania films. The thickest titania film (*i* = 20) without MNLs exhibits the highest nanoparticle coverage and shows an *E*_g_ mode at ∼ 143 cm^–1^ indicative of anatase TiO_2_^50^ (Figure 9a). This signature is not seen in titania/MNL multilayers, consistent with MNL-induced suppression of nanoparticle formation from titania. Furthermore, spectra from high nanoparticle coverage regions show the anatase *E*_g_ mode signature, which is undetectable from low-to-no coverage regions (Figure 9b). These results show that the nanoparticles are crystalline, while the titania film is amorphous.

## 3 Conclusions

We have synthesized multilayers of titania/MNL interfaces by combining ALD of titania and single-cycle MLD of organophosphonates. Our results show that the growth rate of titania nanolayers on the MNLs is significantly lower than that on titania surfaces without an MNL. This MNL-induced growth rate suppression is greater for aliphatic-backboned PDDP than for the aromatic-backboned PBPP. Similarly, the interfacial MNLs also suppress anatase TiO_2_ nanoparticle formation from the amorphous titania films, with PDDP having a greater suppression effect. MNL-induced diminution of titania growth rate and suppression of nanoparticle formation are sensitive to the molecular coverage and morphology which are related to the backbone structure. As a consequence, TiCl_4_ decomposition kinetics and Cl trapping at MNL-covered surfaces are sensitive to the MNL coverage and morphology. The roughness of titania/MNL multilayers is greater than that of titania films without MNLs, but bears no direct correlation to the MNL structure, suggesting that the roughening is due to the titania/MNL bonding chemistry. Our results showing MNL-induced effects on the growth kinetics, morphology, and stability of the inorganic nanolayers are valuable for nanomolecularly engineered hybrid multilayered nanolaminates.

## 4 Experimental Details

### 4.1 Inorganic–Organic Hybrid Multilayer Thin Film Synthesis

All our titania/organophosphonate-MNL multilayer films were grown on Si(100) substrates covered with a ∼300 nm-thick oxide layer. Prior to film deposition, the substrates were cleaned by successive rinses in acetone and 2-propanol followed by drying in high purity N_2_. Thermal ALD of titania and single-cycle MLD of the organo-diphosphonate MNLs were carried out in a Picosun R200 Advanced reactor at 180 °C and 700 Pa in a Class-100 cleanroom. For titania ALD, we pulsed TiCl_4_ (99+%) for 0.2 s and deionized water H_2_O (>18 MΩ) for 0.1 s. For single-cycle MLD, we pulsed the MNLs for 120 s from ampules heated to 180 °C. After each ALD and single-cycle MLD pulse, we purged the reactor with 120 sccm N_2_ carrier gas for 6s.

### 4.2 Organophosphonate Molecular Synthesis

Tetraethyl-[1,1′-biphenyl]-4,4’diylbis(phosphonate) and diethyl-(12-phosphonododecyl) phosphonate were synthesized from 4,4′-dibromobiphenyl and 1,12 dibromododecane using triethylphosphite via a Michaelis–Arbuzov reaction. TGA and DSC measurements were carried out in Discovery TGA 55 and Q100 DSC instruments from TA Instruments, respectively, with heating rates of 10 °C/min. A MBraun LABmaster 130 glovebox filled with 99.998% N_2_ was used for the TGA experiments as well as loading and hermetically sealing DSC pans.

### 4.3 Characterization by Microscopy, Spectroscopy, and Diffraction

Transmission electron microscopy (TEM) was carried out by using a double-tilt holder in a FEI Tecnai T20 ST instrument operated at 200 kV. Cross-section TEM samples were prepared by focused ion beam milling in a FEI Scios dual beam system. Scanning electron microscopy (SEM) was carried out in a Zeiss SUPRA 55 FESEM system at 12.5 keV using an in-line secondary electron detector with a stage tilt of 10° unless specified otherwise.

X-ray photoelectron spectroscopy (XPS) was carried out in a PHI 5000 VersaProbe system with ∼10^–8^ Pa base pressure. The spectra were analyzed using MultiPak software with background subtraction by an iterative Shirley method and charging-related energy shifts corrected with respect to the adventitious C peak.^38^ All measurements used a 23.5 eV pass energy, except for the PBPP P 2p band spectra (Figure 2b) which required a 46.95 eV pass energy to obtain a clear signal. As-received organophosphonates were analyzed by XPS by applying small drops of liquid PDDP or drop-casting a solution of PBPP in toluene on a Si substrate. XRR measurements were carried out in a PANalytical X’Pert Pro X-ray diffraction system using a graded parabolic X-ray mirror beam conditioner. XRR data was fit using the PANalytical X’Pert Reflectivity software.^43^

Rutherford backscattering spectrometry (RBS) and Elastic recoil detection analysis (ERDA) were carried out at the Tandem Laboratory in Uppsala University.^51^ For RBS, a 2 MeV ^4^He^2+^ beam was used incident at 5° to the surface normal with the detector placed at a 170° scattering angle. The data was calibrated with Au, Ni, Si, and Cu standards, and fit using SIMNRA^46,47^ simulations. In ERDA, the 36 MeV ^127^I^8+^ probe beam was incident at a 22.5° angle to the surface, and a time-of-flight detector was set at a 45° angle to the surface. The data was calibrated with Al_2_O_3_, Au, CaF_2_, Mo, SiC, and TiN standards, and analyzed using Potku^48,49^ software. Raman spectra were acquired using a Horiba Jobin Yvon Raman spectrometer (HR Evolution) with a 633 nm laser excitation source. The spectra were recorded using a 50x objective lens and 1800 grooves/mm grating with a Peltier cooled (CCD) detector.

## Supporting Information Available

Elemental composition ratios of titania surfaces exposed to PDDP from XPS; electron diffraction analysis of TEM cross sections for films of titania multilayers with and without MNLs; modified Bragg’s law used to calculate periodicity from XRR Bragg peak positions (PDF)
