# Supplementary material for: Nanomolecularly-induced Effects at Titania/Organo-Diphosphonate Interfaces for Stable Hybrid Multilayers with Emergent Properties
Source: ACS Appl Nano Mater. 2024 May 3;7(10):11225–33. doi: 10.1021/acsanm.4c00743 (PMC11129189; doi:10.1021/acsanm.4c00743)
Supplement: Supplementary file 1 — an4c00743_si_001.pdf [file an4c00743_si_001.pdf]

Supporting Information

Nanomolecularly-Induced Effects at Titania/Organo-Diphosphonate Interfaces for Stable Hybrid Multilayers with Emergent Properties

Collin Rowe[1], Ankit Kashyap[2], Geetu Sharma[1], Naveen Goyal[3], Johan G. Alauzun[4], Seán T. Barry[5], Narayanan Ravishankar[3], Ajay Soni[2], Per Eklund[6], Henrik Pedersen[6], Ganpati Ramanath[1,6*]

[1]*Materials Science & Engineering Department, Rensselaer Polytechnic Institute, Troy, NY 12180*

[2]*School of Physical Sciences, Indian Institute of Technology Mandi, Mandi, Himachal Pradesh 175005, India*

[3]*Materials Research Centre, Indian Institute of Science, Bangalore, Karnataka 560012, India*

[4]*Institut Charles Gerhardt, University of Montpellier, CNRS, ENSCM, Montpellier 34293, France*

[5]*Department of Chemistry, Carleton University, Ottawa, Ontario K1S 5B6, Canada*

[6]*Department of Physics, Chemistry, and Biology, Linköping University, Linköping SE-58183, Sweden*

*Corresponding author email: ganpati.ramanath@liu.se, ganapr@rpi.edu

Composition of titania surfaces exposed to PDDP

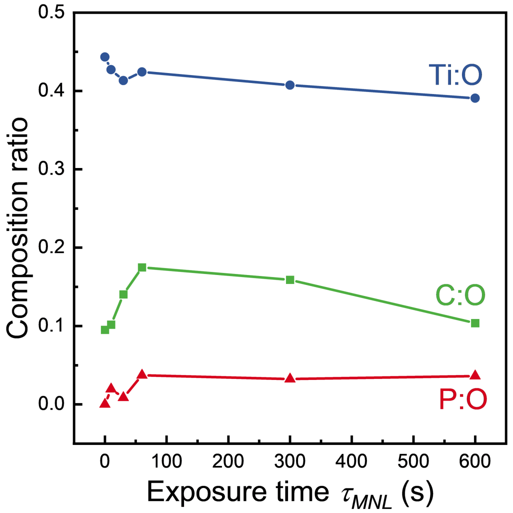

Fig. S1. Elemental ratios determined by normalizing the XPS peak areas of Ti 2p, C 1s, and P 2p with that of the O 1s peak, for titania surfaces exposed to PDDP for 0 s ≤ τ_{MNL} ≤ 600 s.

Diffraction analyses from amorphous titania

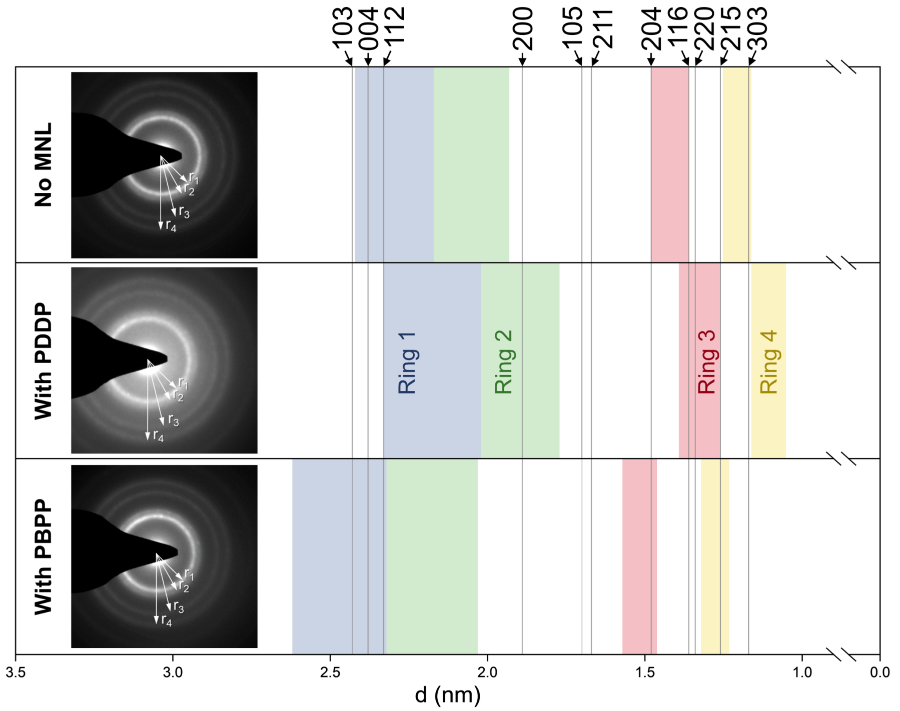

Fig. S2. Comparison of ring positions in TEM diffractograms obtained from titania/MNL multilayers with PBPP, PDDP, and no MNLs, with i = 20. The hkl values for bulk anatase[1] TiO_2 specified by $I4_1/amd$ and lattice parameters a = 0.3785 nm and c = 0.9514 nm, are overlayed for reference.

Multilayer periodicity from XRR

From the Bragg peak positions (e.g., in Fig. 4b), multilayer periodicity is determined from a modified Bragg's law that accounts for refraction through the film: $\lambda = 2d\sin\theta\sqrt{1+\frac{\bar{n}^2-1}{\sin^2\theta}}$, where λ is the X-ray wavelength, d the multilayer periodicity, θ the Bragg peak, and the average refractive index $\bar{n} = 1-\sqrt{2\theta_c}$, where the total-external reflection angle θ_c is indicated by $dI/d\theta_c$ minimum.

Supplemental Reference

(1) Rohrer, G. S. *Structure and Bonding in Crystalline Materials*; Cambridge University Press, 2001. DOI: 10.1017/CBO9780511816116.
